# Improving Throughput for Mid-acuity Patients in the Pediatric Emergency Department

**DOI:** 10.1097/pq9.0000000000000302

**Published:** 2020-05-26

**Authors:** Asha S. Payne, Kathleen M. Brown, Deena Berkowitz, Jeanne Pettinichi, Theresa Ryan Schultz, Anthony Thomas, James M. Chamberlain, Sephora N. Morrison

**Affiliations:** From the * Emergency Medicine and Trauma Center, Children’s National Hospital; † Vapotherm, Inc, Exeter, NH.

## Abstract

**Purpose:**

Visits to pediatric emergency departments (EDs) are increasing, leading to overcrowding, prolonged patient wait times, and negative patient experiences. In our system, these prolonged wait times and negative experiences notably impact mid-acuity patients. As such, we sought to decrease their time-to-first-provider from 92 to 60 minutes.

**Methods:**

After identifying inefficiencies in patient arrival, triage, and assessment, we redesigned our physical space and implemented a new triage process. Further, we deployed a new multidisciplinary front-end team consisting of a physician, nurses, and ED tech specialists to create and implement an initial management plan. Time-to-first-provider for mid-acuity patients was the main outcome measure. We examined ED length of stay (LOS) as a balancing measure. Post hoc, we measured time-to-first-nursing assessment and the proportion of high-acuity patients seen within 20 minutes as additional measures of the impact of these interventions on our system. All analyses were measured using statistical process control charts.

**Results:**

During high patient volumes, we decreased the time-to-first-provider to 70 minutes, but exceeded our goal during low patient volumes (41 minutes). We observed a 5% decrease in LOS during both high and low patient volumes (5% and 8%, respectively). There was a 60% increase in the time-to-first-nursing assessment.

**Conclusions:**

A new front-end process resulted in improved time-to-first-provider and LOS. The new process was associated with longer times for nursing assessments but did not negatively impact the rapid physician assessment of higher acuity patients.

## Abstract 2

Supplemental Digital Content is available in the text.

## INTRODUCTION

Emergency department (ED) visits are increasing for the pediatric population.^1–3^ With rising patient volumes,^2,3^ pediatric EDs face overcrowding and prolonged patient wait times. ED overcrowding threatens patient safety, negatively impacts patient experience, and creates a risk of clinical deterioration or prolonged pain.^4,5^

Several ED throughput improvement studies focus on early processes in the ED course to mitigate the impacts of overcrowding.^5–7^ One institution attempted to decrease ED length of stay (LOS) by increasing efficiency with front-end processes such as in-room registration,^7^ while another trialed immediate bedding to decrease door-to-provider (physician or nurse practitioner) time and improve the patient experience.^5^ Rutman et al^8^ sought to redesign their front-end processes by using a registered nurse in the lobby and direct patient rooming: immediate, team-based assessment and the use of an early initiation team to begin orders when the primary team was not available.

The Emergency Severity Index (ESI) defines patient acuity on a scale of 1–5, with ESI 1 as the highest acuity, unstable patients needing to be seen immediately.^9^ The lowest acuity patients, ESI 5, are stable patients who can wait to see a provider and will not require ED resources.^9^ Mid-acuity patients (ESI 3) are stable but require ED resources. During our busiest periods, bed shortages adversely affect patient arrival-to-provider times, particularly for mid-acuity patients, which represent approximately 30% of our annual visits. These patients are negatively impacted by overcrowding and face longer wait times and delays in care than their more acute counterparts. Paradoxically, because we have separate processes for low-acuity patients who often do not require beds, our ESI 3 patients often have longer wait times. We performed this quality improvement effort specifically to decrease the time-to-first-provider for mid-acuity patients. Our primary aim was to decrease the time-to-first-provider from our baseline of 92–60 minutes.

## METHODS

Children’s National Hospital ED is an urban, academic, tertiary care, Level 1 pediatric trauma center located in a free-standing children’s hospital. Board-certified pediatric emergency medicine (PEM) physicians, fellows, general pediatricians, advanced practice providers, and residents staff the ED. At our institution, “provider” is either a physician or an advanced practice provider. Additional clinical staff include ED nurses, ED tech specialists, and interpreters. The ED and waiting room accommodate 45,000 visits per year, but our annual volumes exceed 87,000 visits.

In July 2016, we conducted a Kaizen event, an intense, week-long improvement session to perform process mapping and plan PDSA (Plan-Do-Study-Act) cycles to improve quality and safety. The team of stakeholders included ED physician and nursing leadership, staff nurses, nursing educators, ED tech specialists, and staff from patient registration, security, and patient experience. Focusing on front-end processes, we identified several inefficiencies regarding patient arrival, registration, triage, and assessment (Table 1). We refined the concept and function of the front-end team and created a strategic plan to renovate the front-end space, as defined by the waiting room, triage, and nurse assessment areas.

**Table 1.**
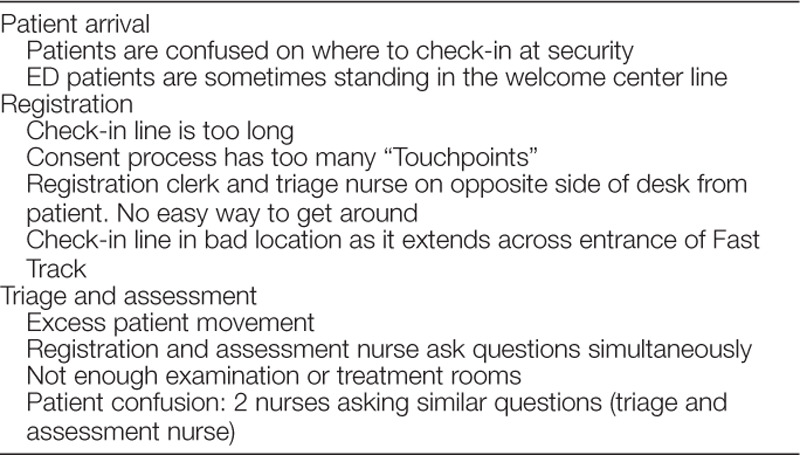
Inefficiencies Identified during Kaizen

**Fig. 1.**
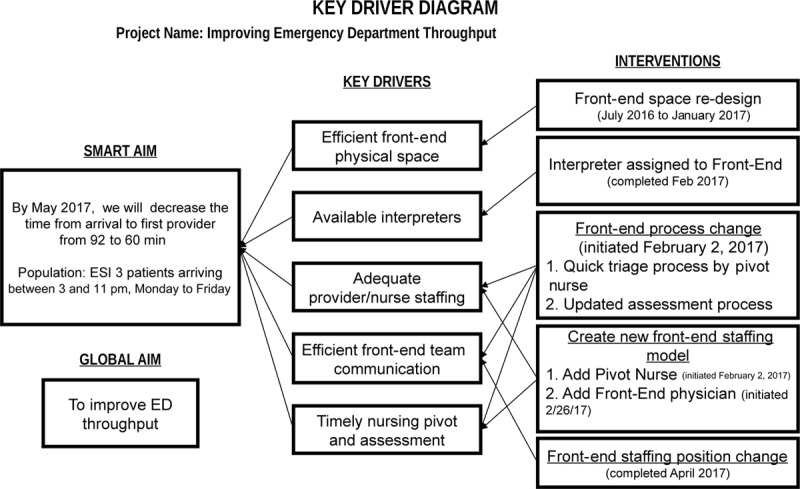
Key driver diagram.

In late 2016, we applied improvement science methodology to study the impact of our proposed changes. The key stakeholders convened to build a key driver diagram (Fig. 1) to display the project aims and theories related to improvement efforts and to track interventions.

### Interventions

In tandem with our efforts to improve the ED flow of low-acuity patients (ESI 4 and 5),^10^ we implemented 2 major interventions to improve the flow of ESI 3 patients: (1) physical space redesign and (2) front-end process change. The design of these interventions occurred in parallel, but the implementation of the new processes could not proceed until the space redesign was complete.

#### Physical Space Redesign

We renovated our waiting room to improve the registration, triage, and assessment processes. The renovation increased the number of nurse assessment rooms from 4 to 7, and patient registration windows from 1 to 2. This change created 2 areas which could accommodate the updated registration and rapid triage (pivot) process outlined below. The resultant changes decreased the size of the waiting room by half but increased the number of front-end patient care rooms. Construction for the physical space redesign occurred from July 2016 to January 2017.

#### Implementation of Front-end Process

The new front-end process had 2 components: (1) a new rapid triage (“pivot”) process and (2) a new multidisciplinary front-end team. Before the intervention, patients waited in line for a single registration clerk, then provided comprehensive clinical information to a single triage nurse, followed by a comprehensive nursing assessment, including vital signs, medication reconciliation, and required screenings. There was often unnecessary redundancy in the information provided to the triage and assessment nurses. After the intervention (February 2017), patients undergo quick registration by a registration clerk, which includes only essential, limited demographic information. Almost simultaneously, patients receive a rapid triage by the “pivot nurse.” When faced with high volumes of incoming patients, the nurse quickly pivots back and forth between patients undergoing registration at the 2 patient registration windows. The rapid evaluation by the pivot nurse captures a limited pertinent medical history and presenting complaint, allowing the assignment of an ESI triage level. In short, the pivot nurse performs a quick triage and assignment of patient acuity. To ensure an expeditious process for our Spanish language patients, we assigned a Spanish language interpreter to the front-end area (February 2017). After pivot, the assessment nurse obtains additional medical information and performs a thorough nursing assessment.

#### Creation and Function of the Front-end Team

Before implementation, we created job descriptions and job aids and provided education for all members of the front-end team. Table 1 (Supplemental Digital Content, available at http://links.lww.com/PQ9/A188) represents the job aid created for physicians. The front-end team consists of a front-end physician, either a subspecialist in PEM or senior pediatrician, a front-end flow coordinator (nurse), assessment nurses, and an ED tech specialist. To maximize communication, we co-located all members of the front-end team. After the pivot process, the front-end flow coordinator facilitates the movement of the patient from pivot to assessment rooms, where the assessment nurse obtains vital signs and a weight, documents the medication reconciliation and allergy profile, and completes all required patient screenings. The goal is for a simultaneously focused history and physical by the front-end physician during the nursing assessment, but this may not occur simultaneously depending on the front-end physician’s workload. The front-end physician initiates a preliminary management plan that may include lab testing, imaging, initial medical interventions, and possible redirection to consultant clinics such as dental or ophthalmology. We created a document template in the electronic health record (EHR) to document a brief assessment and preliminary plan, which facilitates communication between the front-end physician and the remainder of the ED team. Further, we assigned the front-end physician a designated phone number, so all team members had direct communication as needed. An ED tech specialist obtains intravenous access and laboratory studies, if needed, and escorts patients to other care areas. The front-end flow coordinator facilitates patient flow and assists in executing the preliminary management plan (eg, administering medications).

A detailed history and physical examination and interpretation of laboratories and imaging initiated by the front-end team is completed by residents, advanced practice providers, PEM physicians, or ED pediatricians in the routine patient care rooms of the ED, once available. The front-end team is operational during periods of maximum patient arrivals, 3–11 pm, Monday to Friday. The front-end team ran continuously from February 2017 to June 2018. It resumed operations in September 2018 through May 2019, focusing on the higher patient volume.

### Measures

Our specific aim was to decrease the time-to-first-provider for ESI 3 patients from 92 to 60 minutes within 5 months. As a process measure, we also measured changes in time-to-first-nursing assessment after arrival. The preintervention period was defined as the triage-to-nursing assessment; the postintervention period was the pivot-to-nursing assessment. We measured overall LOS for ESI 3 patients as a balancing measure because the front-end process might have resulted in unnecessary testing based on the brief history and physical examination. As an additional balancing measure, we examined the proportion of ESI 2 patients seen within 20 minutes of arrival to assess the possible impact of our interventions on other ED populations.

### Data Collection and Analysis

The analyses of the outcome measure, the LOS balancing measure, and post hoc analysis of time-to-first-nursing assessment were limited to ESI 3 patients arriving from 3 pm to 11 pm, Monday to Friday. Similarly, we limited the analysis of the proportion of ESI 2 patients seen within 20 minutes to 3 pm to 11 pm, Monday to Friday. We excluded patients transferred from other hospitals. Data were retrospectively extracted from the EHR each week (Cerner Corporation, Kansas City, Mo.). We excluded patients with missing data from the analysis for all measures. Also, patients with an arrival-to-first provider time or an LOS greater than the weekly 95% were excluded from the analysis because they represent human error.^10^ Because seasonal variation affects patient volumes, we separately analyzed high-volume (September to May) and low-volume months.

Statistical process control charts, specifically X bar S charts, were used to analyze time measures, and we used a P chart for the proportion of ESI 2 patients seen by a physician within 20 minutes of arrival. We created all statistical process control charts with Minitab 17 Statistical Software (2010) (Minitab, Inc. State College, Pa.). The baseline period was July 2016 through February 2017. Standard criteria identified special cause variation.^11^ An accompanying centerline shift and recalculation of control limits occurred if there were special cause variation and an associated change related to the system. This work is quality improvement and was approved as exempt from review by the institutional review board.

## Results

### Impact of Front-end Team during Higher Patient Volumes

**Fig. 2.**
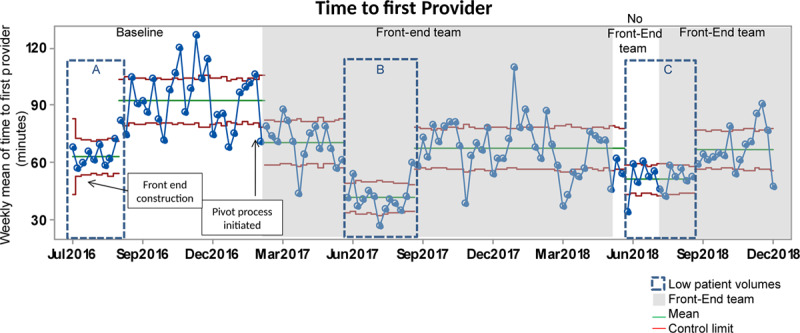
Time-to-first-provider. The baseline period is indicated in white. Upper and lower control limits are in red. The centerline (mean) is in green. The gray shaded areas occurring after the baseline were when the front-end team was active. The Summer of 2018 is white to demonstrate that we did not use the front-end process during this time. The dotted rectangles depict summers with low patient volumes.

During the high-volume periods, the baseline mean time-to-first-physician for ESI 3 patients was 92 minutes, which improved to a mean of 70 minutes after the introduction of the front-end team in February 2017 (Fig. 2). This improvement was sustained at 67 minutes in September 2017, with an additional improvement to 63 minutes in September 2018.

**Fig. 3.**
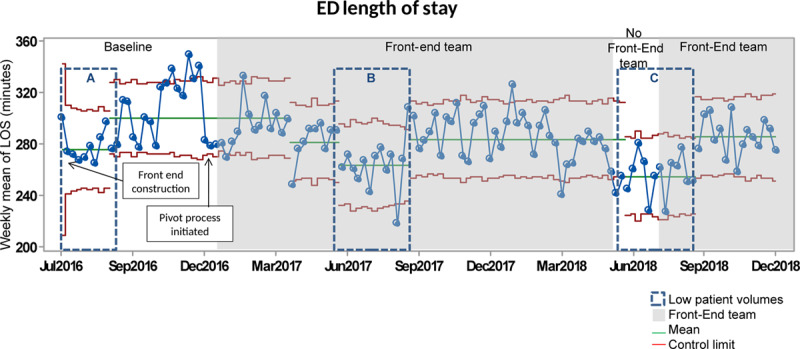
ED length of stay. The baseline period is indicated in white. Upper and lower control limits are in red. The centerline (mean) is in green. The gray shaded areas occurring after the baseline were when the front-end team was active. The Summer of 2018 is white to demonstrate that we did not use the front-end process during this time. The dotted rectangles depict summers (low patient volumes).

Mean LOS was 301 minutes during the baseline period for ESI 3 patients. After implementation, special cause variation was noted, with a 5% decrease in LOS to 282 minutes (Fig. 3), with sustained improvement during the remaining higher patient volume periods.

### Impact of Front-end Team during Lower Patient Volumes

Summer 2016 showed a mean time-to-first-provider of 62 minutes (Fig. 2, box A), decreasing to 41 minutes after implementation of the front-end team in 2017 (Fig. 2, box B). Removal of the front-end team in summer 2018 was associated with an increase in the time-to-first-provider to 52 minutes (Fig. 2, box C).

ED LOS was 276 minutes during the Summer of 2016 (Fig. 3, box A). The introduction of the front-end team decreased LOS to 264 minutes in Summer 2017 (Fig. 3, box B). Removal of the front-end team in Summer 2018 was associated with an additional decrease to 255 minutes (Fig. 3, box C).

### Supplemental Analyses

**Fig. 4.**
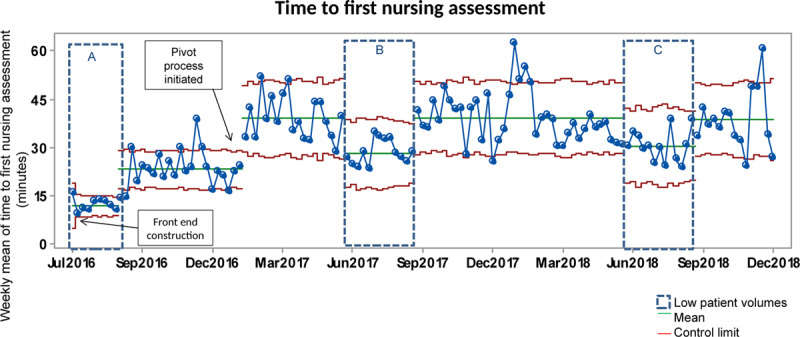
Time-to-first-nursing assessment. Upper and lower control limits are in red. The centerline (mean) is in green. The dotted rectangles depict summers (low patient volumes).

**Fig. 5.**
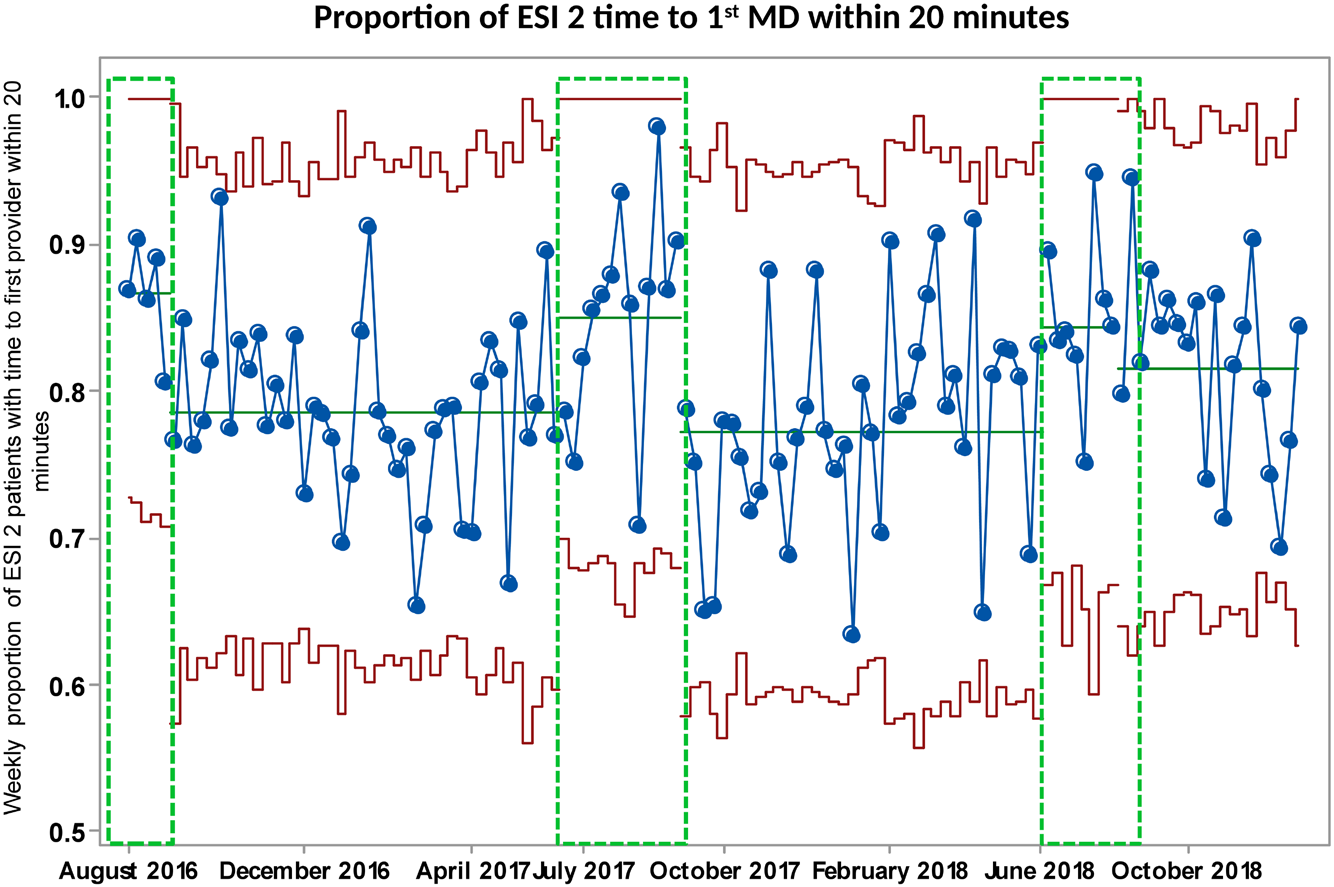
Proportion of ESI 2 patients seen by the provider within 20 minutes. Upper and lower control limits are in red. The centerline (mean) is in green. The green dotted rectangles depict summers (low patient volumes).

The baseline mean time-to-first-nursing assessment during the high-volume period was 23 minutes. After the initiation of the new pivot process in February 2017, we noted special cause variation related to this process change, with an increase to 38 minutes (Fig. 4). The remaining higher volume seasons sustained this change. This increase also occurred during lower volume seasons (27 and 30 minutes, respectively) (Fig. 4, boxes B and C). The proportion of ESI 2 patients seen by a physician within 20 minutes during this period remained stable (Fig. 5), exhibiting the same seasonality as the other measures.

## DISCUSSION

### Summary and Interpretation

By repurposing our waiting room space for additional assessment rooms and implementing a new front-end process, we decreased the time-to-first-physician to 70 minutes, with improvements in both the high- and low-volume baseline periods. ED LOS improved despite the concerns that unnecessary testing ordered in the front-end might lead to longer stays. The time-to-first-nursing assessment increased with the institution of the pivot process, failing to reach the baseline mean, even during lower patient volumes.

To our knowledge, we are the first pediatric ED to publish work regarding the creation of a front-end team, specifically for ESI 3 patients. In analyzing our baseline throughput data, it was clear that we needed to address this mid-acuity subgroup of patients because care was being delayed in comparison to both the more acute and less acute patients. Our findings build upon the work of Partovi et al,^6^ who found that putting a faculty member in the triage area shortened LOS. Our improvement work differed in that we executed this intervention in a pediatric ED, had a longer trial, and created a team to support the interventions initiated by the physician. We also created comprehensive education and job aids for the front-end team. Our work focused specifically on ESI 3 patients, in contrast to other studies that uniformly applied their intervention to all triage categories.^5–8^

### Impact of the Interventions

While we anticipated the improvement in our primary outcome, we did not anticipate the adverse impact on time-to-first-nursing assessment. In analyzing changes to our workflow, implementing the front-end team shifted several tasks from both the triage and bedside nurses (seen later in the ED course, after assessment) to the front-end assessment nurse, thereby delaying the nurse’s ability to assess patients rapidly. Similarly, with this new process, we pivot patients very close to their ED arrival. Before these interventions, patients spent longer in the line awaiting triage, particularly during our high-volume times. The combination of rapidly triaging patients closer to their actual arrival to the ED and shifting additional tasks to the assessment nurse likely increased the time-to-first-nursing assessment.

There were unintended consequences of our interventions. Adding an additional physician in the patient’s ED course created the need for a physician handoff. We did not encounter safety issues as the EHR documentation and designated front-end physician phone limited communication barriers between the front-end physician and the remainder of the ED team. Also, we found that some patients, despite their ESI designation, did not require additional physician management beyond the history obtained by the front-end physician. For example, patients requiring laceration repairs or consults who provide definitive management of the presenting issue (eg, dental) were often managed by the front-end physician without requiring a handoff. This observation was an unanticipated additional benefit of the new process. Finally, decreasing the size of the waiting room created crowding, particularly in high-volume times. The front-end team, initially located in “off stage” areas away from patients, transitioned to working in “on stage” areas of the waiting room area to improve team communication and efficiency. We believe the benefits of an expeditious patient evaluation by a physician and decreased LOS outweighs the negative experience in a crowded waiting room.

### Limitations to Generalizability

There are costs associated with implementing the interventions noted in this study. Teams will need to weigh the capital costs of an ED redesign and staffing of a front-end team against the opportunity to shorten ED LOS and improve the patient experience. Institutions with limited physical plant space and limited funding for the capital outlay should consider re-allocating nursing and physician staff and repurposing other sections of the ED to create a front-end team. Limiting these interventions to high-volume periods mitigates additional staffing costs.

We acknowledge both the special cause variation and seasonality present in the data as a limitation of our work. Although trends in patient arrivals based on time of day or season (eg, more arrivals in the winter, less during the summer) can be predicted, more minute variations cannot. Analyses to account for highly skewed data neither corrected nor eliminated this variation. We chose not to exclude these points as they truly reflect the performance of our system, not outliers caused by inaccurate data. In our system, this special cause variation is evidence that some patients receive a greater impact of the intervention; there is a subset of patients with very short time-to-first-provider and LOS. Some of the variability beginning in late 2018 can be attributed to a larger pool of physicians filling the front-end shift. We may have lost some physician performance consistency due to decreased oversight.

## CONCLUSIONS

These interventions represent a creative use of space and personnel in a pediatric ED with insufficient space for the volume of patients. Other institutions can easily adapt parts of these interventions to meet their needs. The change in initial nursing assessment (pivot) is hardwired into our system. Still, the institution of a front-end provider is turned on and off to meet the needs of our patient volumes, thus creating a sustainable intervention. Rapid evaluation by a physician early in the patient encounter can be applied to other EDs and other high-volume clinical settings. Further study should include a direct measurement of these interventions on the patient experience.

## DISCLOSURE

The authors have no financial interest to declare in relation to the content of this article.

## Supplementary Material


