# Supplementary material for: Improving Throughput for Mid-acuity Patients in the Pediatric Emergency Department
Source: Pediatr Qual Saf. 2020 May 26;5(3):e302. doi: 10.1097/pq9.0000000000000302 (PMC7297400; doi:10.1097/pq9.0000000000000302)
Supplement: Supplementary file 1 [file pqs-5-e302-s001.docx]

Supplemental table 1. Front-End Physician Job Aid

| **Task** | **Key points** | **Rationale** |
| --- | --- | --- |
| Obtain an phone and let key staff know your number  Phones located in lockbox | Write your number on the ED white boards on all 4 sides  Give your number to the flow nurse and charge nurse | You need to be able to communicate with front-end and back-end staff |
| Communicate your presence to back-end staff | Talk to charge physician and charge nurse and let them know you will be performing intake function and your hours  Specifically communicate that you will be signing in to some patients as **INTAKE MD ####** but that they still need to see these patients and that they can call you if they have questions | Ensure all staff are aware of the process and expectations  Avoid unnecessary patient care delays due to poor communication |
| Huddle with all members of the front-end team | Identify roles and review the process  Primary   - Initiate ESI 2 Powerplans if no direct bed available in back then move to SWR - Start work up for ESI 3 patients eg. abd pain. - Wound care not requiring sedation - Identify next location for patient, bed, SWR/RSU, WR - Identify specific consults eg. ophthalmology, dental and page - Identify Pre-arrivals needing specific testing or evaluation - Evaluate sepsis trigger patients - RN triage resource   Secondary   - See low acuity patients if no high acuity in queue | Situational awareness and shared mental model (of the process) |
| Obtain a Computer on Wheels (COW) or tablet that can print to front end | Make sure COW prints to appropriate area | You will need to sign in for patient and write orders.  You need to be mobile |
| Screen the tracking board for patients that may benefit from front-end team | Look for patients who may need:  Pathway initiation  Pain medication  Labs studies  Radiographs  Specific Pre-arrival requests  Referral to subspecialty clinic (dentistry, ophthalmology, etc.) | Expedite care in patients who will need these resources |
| Screen identified patients in person | To be done in front-end assessment room with or after RN assessment | Avoid duplication of questions  Allows for relative privacy and avoids unnecessary movement of patient and staff |
| Patients not ideal for front-end physician | Psych without clear suicidal or homicidal ideation or escalation who will need Psych evaluation, police clearance, alleged physical or sexual assault (unless the clinic is open and can take them), medicolegal documentation | These patients usually are quite involved and will likely not allow for a quick assessment and work up |
| Sign in for patient as INTAKE MD #### |  | Visual cue for staff that patient has been screened by intake team |
| Introduce yourself and your role to the family | *“I am DR X and I am here to see if we can get your care started while you are waiting for an available room (or provider)*  *Another provider will be seeing you and doing a more thorough assessment and checking on the tests that I order”* | Set appropriate expectations for care and keep family informed of process and anticipated wait times |
| Perform brief focused History and Physical | Provide estimated wait times for orders you place e.g. 1hour for labs. | Confirm need for resources and appropriateness of orders |
| Document your medical decision making on Front-End Physician form | Front-End Physician form:  Complaint  Key Information (free text)  Labs/Imaging  Next Steps | Non-verbal communication of your thought process to back-end team |
| Write appropriate orders and communicate them to the front-end flow coordinator/ED tech |  | Expedite needed testing, treatments |
| Screen the next appropriate patient | Appropriate patients as listed above  In general low acuity patients are not appropriate for front-end physician  See secondary role if no high acuity for front-end | Goal is to expedite care for patients who will need resources  Providing care to fast track patients who do not need any other resources will prevent you from seeing the next appropriate intake patients |
| When available perform minor procedures | e.g reduce nursemaids elbow | Provide timely care |
| Chart on patients who are appropriate discharges from Front-End (This should be rare) | Appropriate patients may include reduced nursemaid’s elbow, laceration that does not need suture repair  May use scribe if available for documentation | This should be kept to a minimum to avoid missing the next patient who would benefit from the Front-End Physician |
| Answer questions about pivot/assessment from the front-end team | e.g Can this patient go to Fast Track?  Does this patient need isolation?  Does this patient have sepsis? | Facilitate accurate triage and placement of patients |
| Answer questions from back-end staff about patients you have seen | This can be minimized by communicating your role with the whole team on arrival | Facilitate timely and safe patient care |
|  | | |
